# Pioneer women in the development of toxicology in Brazil

**DOI:** 10.1590/1414-431X202011177

**Published:** 2021-04-02

**Authors:** L. Melo, T.S. Melo

**Affiliations:** 1 Laboratory of Investigative Toxicology and Pathology, Department of Environmental and Occupational Health, School of Public Health, Indiana University, Bloomington, IN, USA; 2 Centro de Ciências Biológicas e da Saúde, Universidade Presbiteriana Mackenzie, São Paulo, SP, Brasil

**Keywords:** Toxicology, Women in toxicology, Brazil, Women in science, Brazilian scientists

## Abstract

Women have always played an important role in the development of toxicology all over the world. Specifically in Brazil, toxicology has had greater female representation than other countries, but women's participation at high hierarchical levels is low. Although more than 62% of the members of the Brazilian Society of Toxicology are women, only 7 out of the 22 presidents have been women throughout its 48 years of existence. This article aims to celebrate women in the field of toxicology in Brazil, based on interviews with five of these scientists who have changed the field of toxicology in Brazil as we know it today, each in their specific sub-areas. These women are: Dr. Ester de Camargo Fonseca Moraes, Dr. Silvia Berlanga de Moraes Barros, Dr. Alice Aparecida da Matta Chasin, Dr. Gisela de Aragão Umbuzeiro, and Dr. Tania Marcourakis. They are not only pioneers but they are also examples of admirable persistence in fighting the adversities presented to them. They broke the glass ceiling and opened doors for future generations of women in science. We hope that this article helps inspire women in their careers in toxicology.

## Introduction

Women have always played an imperative role in the development of the field of toxicology worldwide. As a matter of fact, the earliest record in toxicology (4000 BC) involved the “Goddess of Healing” and many of the first toxicology practitioners were female poisoners (1).

More specifically in Brazil, general toxicology is known since the times of the native people. However, as a research and teaching area, it arises in 1966 when Dr. Ester de Camargo Fonseca Moraes implemented Toxicology as an autonomous field in the Pharmacy School at the University of São Paulo. In 1972, the Brazilian Society of Toxicology (SBTox) was founded with the intention of uniting professors, professionals, companies, and organizations interested in the development of toxicology in the country. Four years later, the first Brazilian scientific event in toxicology, the “1st Congress of Tropical Toxicology”, took place in Manaus, and in 1979, the first official SBTox Congress, the “1st Brazilian Toxicology Congress”, took place in a costal city in São Paulo state (2).

In Brazil, in particular, toxicology is an area with a large female representation, since most of its professionals begin their careers in pharmacy, biology, or chemistry courses, which are courses with a good percentage, if not a majority, of women. Of the five awarded scientists with the highest title of honor granted by the SBTox, the Ester de Camargo Fonseca Moraes Medal, three are women: Dr. Maria Elisa Pereira Bastos de Siqueira (2011), Dr. Silvia Berlanga de Moraes Barros (2013), and Dr. Alice Aparecida da Matta Chasin (2015) (3).

Despite the high female representation in Brazilian toxicology, one can notice the decline in number and participation of women at higher hierarchical levels (4), as seen in many other workplaces. To this day, due to the still prevailing belief that women and science are somehow incompatible, women still face the glass ceiling preventing them from reaching higher hierarchical positions. While more than 62% of the SBTox members are women, (5) only seven out of the 22 past presidents were females in the 48 years of the organization (6).

Thus, this article, based on a collection of interviews, celebrates the pioneering women in toxicology in Brazil who broke the glass ceiling and played an important role, opening doors for the future generations of women in science.

## Dr. Ester de Camargo Fonseca Moraes

**Figure 1:**
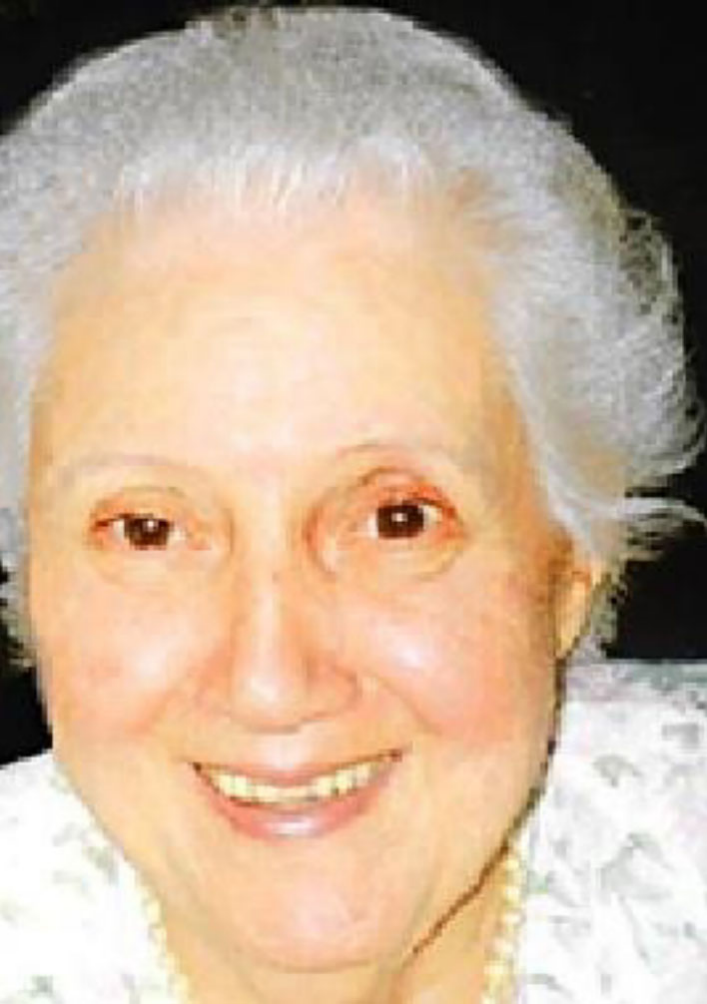
Dr. Ester Moraes. Courtesy of Dr. Regina Lucia de Moraes Moreau.

Talking about the development of the field of toxicology in Brazil must involve talking about Dr. Ester (Figure 1). All the women celebrated in this article, as well as all the Brazilian toxicologists of the time, were trained by Dr. Ester at some point in their lives.

Born on December 8, 1920 in the city of Paraibuna in São Paulo state, Dr. Ester received her Bachelor of Pharmacy degree at the then-called Pharmacy and Dentistry School of the University of São Paulo (USP) in 1942. During her last undergraduate year, Dr. Ester's great academic performance caught the attention of Dr. Linneu Prestes, who was a Professor in the Department of Toxicological and Bromatological Chemistry. In 1943 she accepted his invitation to be a volunteer assistant lecturer.

The following year marked the beginning of her career. She was admitted into the Department of Toxicological and Bromatological Chemistry as an assistant professor, and as a technical specialist, she accepted the role of assisting chemist in the Chemical Service of the Jockey Club of São Paulo - both with the endorsement of Dr. Linneu Prestes, then the Head of the Chemical Service. Thus, Esther had the chance to pursue both teaching and technical careers integrating the theoretical side of teaching and laboratory practice.

During the 22 years that followed the beginning of her career - 1944 to 1966 - Prof. Ester contributed significantly to the development of toxicology in Brazil. Because of her high technical-scientific rigor and excellent teaching skills, she became the head of both theoretical and practical teaching in Toxicological Chemistry. In May 1951, she applied for a professor position while pursuing her doctoral degree, as allowed by the University of São Paulo regulations at the time. She was appointed the position, being considered highly qualified for the position by all members of the Judging Committee.

Likewise, thanks to her skills, work ethics, and dynamism, she went from a humble one-room facility as an assistant chemist in the Chemical Service to Chief Chemist-Pharmacist to the high-level facilities of the Anti-Doping Control and Research Service in the Jockey Club. During this period, Dr. Ester founded the doping control laboratory, which became the first toxicological research center in the country. The laboratory was well equipped, had highly skilled technical staff, and maintained permanent scientific collaboration with similar entities and universities around the world to keep up-to-date with the requirements of toxicological analysis. Many studies were carried out and the laboratory became a well-established collaborative facility receiving dozens of professionals from various regions of Brazil and other South American countries, such as Argentina, Colombia, and Guatemala in search of training and specialization in toxicology. In 1959, Prof. Ester was admitted, through CV and practical examination, to the Association of Official Racing Chemists (AORC) board, an international scientific entity for the coordination of research and confidentiality related to doping control. From 1958 to 1965, more than 16,000 samples were analyzed by the Service, from computed routine analyses to experimental doping.

The year of 1966 was remarkable in Dr. Ester's trajectory since she headed the genesis of toxicology as an autonomous department. The department’s first days coincided with the transfer of the Pharmacy School to the Butantã campus, also known as University City. Because of this, everything was done from scratch: the acquisition of reagents, glassware, and equipment, the construction of laboratories with the right installation requirements, the hiring of teaching and administrative staff, and, above all, the budget allocation, which was very little. All this had a very high price, which the toxicology field paid for its independence as a department.

To sustain such a high commitment, Dr. Ester made one of the most difficult decisions of her professional life in 1968: she resigned from her 24-year-long position in the Anti-Doping Control and Research Service at the Jockey Club of São Paulo, despite the financial disadvantage and the increased working hours, to take over the now autonomous Department of Toxicology, under a full-time teaching and research regimen (RDIDP in Portuguese acronym) at USP. She dreamt of a similar success in the Toxicology Department as she had achieved at the Jockey Club; that is, to found a toxicology school in the country.

The Anti-Doping Jockey Club lab took a lot of her time, but on the other hand, she knew that it was impossible to establish an entirely new department having only one professor and some vials with a few grams of alkaloids. Thus, Dr. Esther began her work as RDIDP, and with the courage and help from many, the battle was gradually won.

All her efforts were rewarded by the end of 1969, when the Department of Toxicology equated the old departments in the School, making Dr. Ester a pioneer in the implementation of toxicology as an autonomous entity. Later, the discipline was also instituted in other Pharmacy Schools in Brazil in public and private universities, active to this day.

During the period between 1968 to 1970, university reform, instituted during the military dictatorship, produced a new paradigm of higher education in Brazil. This reform consisted of a series of regulations that resulted, among other measures, in the merging of small departments into larger ones aiming to integrate teaching and research of related disciplines. With the university reform, the Department of Toxicology became part of the newly created Department of Clinical and Toxicological Analyses of the Pharmaceutical Sciences School, of which Prof. Esther was also the chair. Right after that, in 1972, she was promoted to full professor of Toxicology, and as a result of the reform, she was also integrated into the Department of Clinical and Toxicological Analyses.

On this occasion, Dr. Esther proposed to establish a toxicological laboratory, parallel to the clinical laboratory in the Department. In 1971, funds were authorized for the minimum and sufficient installation of the Toxicological Laboratory. Thus, the Laboratory of Toxicological Analyses (LAT) was implemented and, with time, became a renowned lab within higher education in Brazil.

The LAT was created with the broad goal of being not only a service provider but also a training center. The LAT's leadership was also present in the development and implementation of analytical techniques associated with toxicological diagnosis, associating teaching, research, and extension in the various areas of toxicology.

Under Dr. Ester's leadership, the LAT became a pioneer in Brazil in conducting urine analyses to control doping in athletes, starting in 1974, with a contract signed with the São Paulo Football Federation. The LAT was also responsible for controlling doping in some international events (7) such as the “World Youth Volleyball Championship” (1977), “Pan American Junior Cycling Championship” (1980), “South American Women's Volleyball Championship” (1981), “World Youth Fencing Championship” (1987), “International Cycling Round of Brazil” (1987 and 1988), and “International São Silvestre Run” (1990 and 1991).

Dr. Ester consolidated her leadership in toxicology by creating the first *stricto sensu* graduate course in the field in the country, named Master’s Program in Toxicological Analyses in 1972 and Doctorate Program in Toxicology in 1978, which trained many masters and PhDs responsible for the expansion of toxicology throughout the country, of which some we tell the story here.

Dr. Ester retired in 1987 from the professor position after 44 years of full and uninterrupted activity, continuing as a voluntary advisor for master and doctoral students for another 5 years. In 2002, she received the “Pharmaceutical Merit Commendation”, awarded to members representing the pharmaceutical entity from the Federal Council of Pharmacy, for the relevant services provided to the pharmaceutical profession and to Brazil’s Pharmacy, in general. In the same year at the National Meeting of Graduate Studies in Pharmacy, she was honored for her important and decisive role in implementing the graduate program of the Pharmaceutical Sciences Department of USP (7).

In 1991, Dr. Esther, together with Dr. Nilda G. de Fernícola and Dr. Rywka B. Sznelwar, published the first book of the field in Brazil “Manual of Analytical Toxicology”, which represented the efforts made from the beginning of the graduate program in 1972. The book describes specific methods of analysis of 56 substances based on extractions from dissertations and theses defended in the department during the period (8).

In 2011, the Brazilian Society of Toxicology instituted the “Dr. Ester de Camargo Fonseca Moraes Medal” to honor professionals who have provided services or contributed considerably to the development of toxicology in Brazil. Dr. Ester's name was chosen in recognition of her relevant participation in consolidating toxicology as an autonomous science in the country. As a sign of her importance, her name was mentioned in all the interviews with the toxicologists of this article, designated as an “idol”, a “master”, a “strong person”, who confronted many people, mostly men, to conquer what she believed was necessary for the toxicology field in Brazil. In 2014, the members of the 10th Latin American Meeting of The International Association of Forensic Toxicologists (TIAFT) selected her name as title of the award certificate for one of the best oral papers presented during the event (7).

Dr. Alice Chasin says she holds an immense affection for Dr. Ester for her support in pursuing her studies despite having children. Dr. Alice states that Dr. Esther was always very understanding about the difficulty of balancing career and family and shared that Dr. Ester herself had similar difficulties.

Dr. Esther said she had no restrictions for being a woman beyond those she imposed herself. “I had doubts about whether my dedication to the academic career would harm my children”, she said in the newsletter 53, CRF-8, but added that “today I think my professional accomplishments has helped me in my role as a mother.” In fact, her daughter, Dr. Regina Lucia de Moraes Moreau, has a PhD in toxicology from USP and currently is a retired professor of the Department of Clinical and Toxicological Analyses. She says she was raised in an environment in which studying and working with dedication have always been considered natural values.

In 1994, when Building 13B of the Pharmaceutical Sciences Department was built, a commemorative plaque in honor of Prof. Esther was installed as recognition and gratitude from all those who pursued a career in toxicology.

## Dr. Silvia Berlanga de Moraes Barros

**Figure 2:**
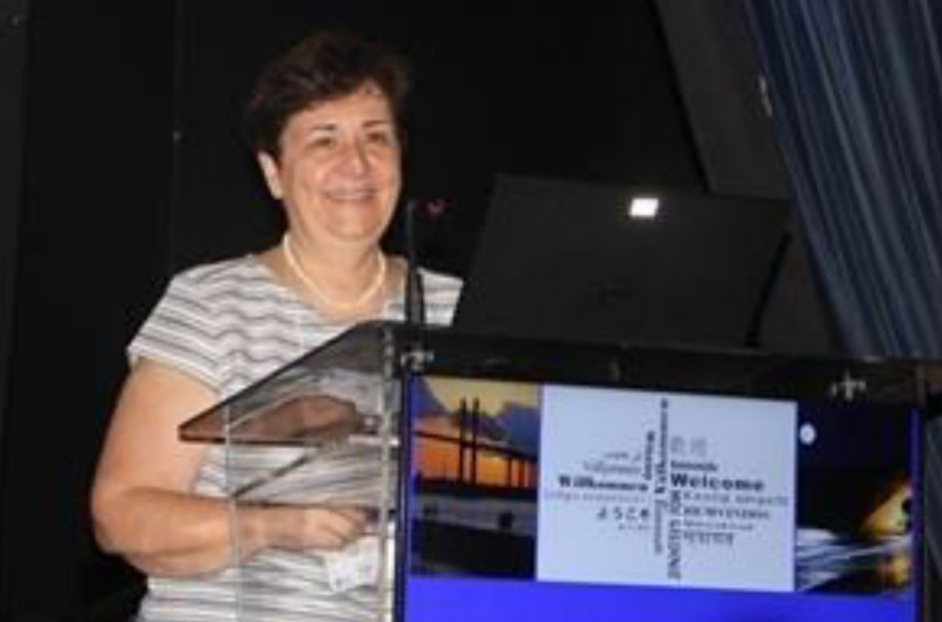
Dr. Silvia Barros at the 9th Congress of Toxicology in Developing Countries and XIX Brazilian Congress of Toxicology. Courtesy of Brazilian Society of Toxicology.

Dr. Silvia (Figure 2) was born in São Paulo and was always encouraged to study by her father who was a teacher and later a professor at USP. Married young, her mother chose to take care of her daughters instead of pursuing a professional career, which was a frustration for her. This frustration was the fuel for her constant incentive for her two daughters to study and always made that a natural part of their lives.

Her father also supported and encouraged his daughters to study. An interesting fact, however, is that he used to say that it was not difficult to raise Silvia and her sister, but this was only because her mother was the one raising them while he worked. Therefore, their mother was the greatest promoter for them getting a higher education and becoming financially independent women. Married for more than 60 years, Silvia's father and mother were strict with education, and after her father became a professor at USP, the university was always an extension of their home.

As a child, Silvia had the opportunity to study in a private school, the Dante Alighieri Middle and High School, which gave her a great scientific basis. In 1966, her sister, who was 2 years older than her and for whom Silvia had great affinity and admiration, was taking entrance exams for medical school. As she was not accepted, she enrolled at the Pharmacy School at USP, the most common second option for those aiming medicine at that time.

During high school, Silvia was fascinated by engines and was considering to become an engineer, but physics was not her strong suit. Because of her affinity with her chemistry professor and her passion for the subject, she changed her mind into studying chemistry. However, seeing how much her sister enjoyed the Pharmacy School, in 1967, Silvia decided to study Pharmacy as well.

She promptly fell in love with the program. In the first year, she was an assistant lecturer in the organic chemistry course, and with the help of great professors, her love for chemistry grew even more.

Silvia was on campus the whole time. During school holidays, she would visit laboratories and spend more time at the university than at home. Coincidentally, Dr. Ester was her neighbor across the street. Another coincidence was having Dr. Alexandre Rossi also as a neighbor. During Silvia’s third year of Pharmacy school, Dr. Rossi was doing his PhD and invited her to work with him in the Clinical Biochemistry Laboratory. She started helping with laboratory maintenance such as washing test tubes and arranging drawers, but with time she started assisting with experiments.

At that time, one of the great toxicology topics was the aflatoxin. The laboratory in which she worked focused primarily on this toxin, especially after the laboratory principal investigator (P.I.) Prof. Durval Nogueira visited England. It is important to emphasize that the technical conditions at that time were not adequate. The same conditions from that time would never be replicated today, and all the researchers, including Dr. Silvia were highly exposed to aflatoxin when producing and isolating it. Regardless of setbacks, her interest in toxicology was growing and by the fourth year of school, she enrolled in the toxicology course.

Following graduation from Toxicology in December 1972, and with Dr. Ester’s encouragement, Silvia applied for the master’s program in Toxicology at USP with Dr. Adayr Saliba as her advisor. Her research focus was on the effects of hexachlorocyclohexane in the liver and her dissertation title was “The Study of Experimental Intoxication by Hexachlorocyclohexane (HCH) in Rats (*Rattus Rattus Norvegicus*)” (9).

In 1974, before completing her master's degree, Silvia was appointed, as at that time, professorships were done by nomination, Assistant Professor in the newly established General Pathology Department at the Pharmacy School.

Dr. Silvia continued her graduate studies and received a doctorate degree in toxicology while being a professor of general pathology, in the same line of research and under the same supervisor, Dr. Salib. Her thesis title was “The Influence of Technical Hexachlorocyclohexane in Liver and Kidney Enzyme Systems in Rats (*Rattus Rattus Norvegicus*)” (9).

Toxicology has always been in her life. Since her hiring by the Pharmacy School, she has been a member of the SBTox, of which she would later become president. All her research work was related to toxicology in some way. She and her sister, Dr. Virginia Junqueira, former professor in the Department of Biochemistry at USP, collaborated intensely on the understanding of the hepatotoxicity mechanisms induced by oxidative stress and published numerous articles together.

At that point in her career, Dr. Silvia was already involved with different toxicology and biochemistry organizations, and in organizing meetings - an activity that has always interested her. Moreover, she always considered important being politically involved in the administrative positions in scientific and academic organizations - not for her own benefit, but always considering what could be improved for future generations. For those reasons, she served as Secretary (1990 and 2004) and President (1992) of the SBTox (6).

Dr. Silvia describes her time at the SBTox as productive but limited given the lack of recognition of toxicology as an independent discipline. Many researchers in various research fields do research in toxicology, but do not consider themselves toxicologists. Thus, many toxicologists do not join SBTox, because they do not see themselves as part of this research field.

In 2000, Dr. Silvia took a further step and became affiliated to the Society of Toxicology (SOT), based in the USA. She was impressed that the SOT united a large number of people working as volunteers for the society's cause and internationalization.

Dr. Silvia has participated in numerous events and committees in the SOT, standing out for her impressive contribution to the Hispanic Organization of Toxicologists (HOT) Special Group since its start (10). Dr. Silvia also devoted herself to the International Union of Toxicology (IUTOX), being part of the board for 8 years now (8). At USP, she held several leadership and management positions at the Pharmaceutical Sciences School and was the Director of Graduate Studies for several years in the Toxicology Department.

Dr. Silvia says that her mother always taught her not to be afraid to be a woman and that being a woman should never be a problem professionally. She managed to raise her two children and have a busy career as a scientist. She says that perhaps the only thing that she would have done if she hadn’t had a family would be to study abroad. Her mother was a great support system for Dr. Silvia's family and she said “half my career belongs to my mother” from all the help.

Dr. Silvia's contributions to science and especially toxicology are immeasurable. She is currently a senior Professor at the University of São Paulo, Associate Editor of the Brazilian Journal of Pharmaceutical Sciences and the Food and Chemical Toxicology Journal, member of the Editorial Board of the Current Opinion in Toxicology Journal, as well as a reviewer of several national and international journals, *ad hoc* consultant for several research funding agencies such as the São Paulo Research Foundation (FAPESP), member of JECFA (FAO and WHO Joint Expert Panel on Food Additives), and consultant for the Chemical Substance Risk Assessment.

Dr. Silvia has won more than 23 awards and titles, among them the Prof. Esther de Camargo Fonseca Moraes Medal of the BSTox. Dr. Silvia has more than 104 published articles, 6 book chapters, and 90 papers published in Congress Annals.

## Dr. Alice Aparecida da Matta Chasin

**Figure 3:**
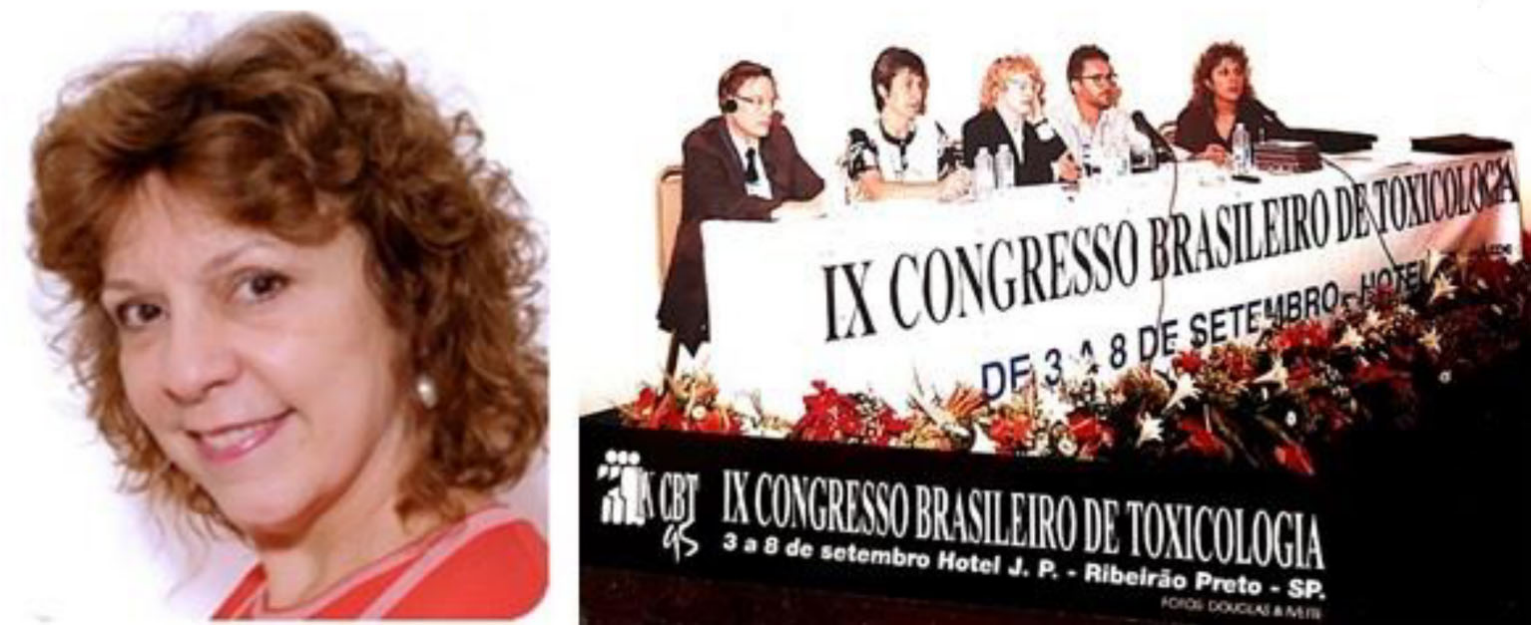
Dr. Alice Chasin (left). Dr. Alice Chasin at 9th Brazilian Congress of Toxicology (right). Courtesy of Dr. Alice Chasin.

Born in São Paulo, Dr. Alice (Figure 3) is the daughter of an Italian mother, from the first generation of Italians in São Paulo, and a military father that met in São Paulo. Since Dr. Alice was a child, she was encouraged to study, which was considered a priority in her family. Dr. Alice describes her father as a great curious man and her mother, even though a housewife, an independent woman, and a fortress that loved life. Coming from an Italian matriarch family in which the grandmother administered everyone’s wages, Alice’s mother did not have the opportunity to study and married when she was 17 years old.

When she was little, she was interested in psychology and wanted to be a psychiatrist when she grew up, but her father always insisted against that. However, her father made sure she studied in a Catholic private school that offered several extracurricular activities, such as theater and piano lessons. The school became a mixed-gender school about the time Dr. Alice started studying there. During her first year in that school, more precisely her fourth year of middle school, the 1964 military coup took place in Brazil and Dr. Alice's father was prosecuted and punished by the military for defending the president João Goulart. Because of this, she had to be moved to a public school, which were excellent in São Paulo, providing her with a high level of education.

For her high school years, she attended the Application School of the Faculty of Education (USP), famous for its political views and excellence, and where many outstanding professionals studied. Dr. Alice’s point of view is that anything that is taught in a fascinating way is eagerly learnt. There, Dr. Alice was able to get closer to science and was fascinated by biology. First, she was considering applying for medicine or biology, but one of her teachers at the school, who was also a professor at USP, advised her to apply for a biochemistry major. Thus, she applied for the Pharmacy and Biochemistry School at the São Paulo State University (UNESP).

From the start, Dr. Alice fell in love with all her coursework, including the interface between toxicology and drug administration. Therefore, the drugs' mechanism of action in toxicology was the bridge between her love for biochemistry and psychology. In her last year as an undergraduate, she started working at the Legal Medicine Institute (IML) of São Paulo.

Although she was satisfied with her job at the IML, she felt like something was missing. At that time, Prof. Dr. Irene Videira de Lima, also a toxicologist and her friend to this day, suggested that she consider an academic career. Even though Dr. Alice always enjoyed pretending to be a teacher when she was little, she says that Dr. Irene “understood my aptitude before I realized it myself”.

Therefore, in 1986, Dr. Alice started a master’s program and needed a supervisor. Despite being the pillar of toxicology at the School of Pharmaceutical Sciences of USP at the time and the perfect fit for Dr. Alice, Dr. Ester already supervised many students and could not accept another one. She suggested other professors to Alice, but all also had too many students. One day, she sat on the School stairs feeling sad and hopeless and afraid of losing the opportunity to pursue a master’s degree when Dr. Antônio Flávio Mídio saw her and asked what was going on. Upon learning the reason for her sadness, he promptly accepted her as a student and was her advisor during all of her master’s and doctorate studies at the Pharmaceutical Sciences School. In 1990, Dr. Alice defended her master's thesis entitled “*Laboratory Diagnosis of Acute Cocaine Intoxication: Forensic Aspects*”, the first thesis written using a computer in the department, and graduated with honorable mention.

During her master's degree, Dr. Alice says that her two idols and great masters were Dr. Maria Aparecida Pouchet Campos and Dr. Ester. An example of this was when in one of Dr. Esther's experimental courses, a class lasted longer than expected and Dr. Alice despaired when she noticed that she had to pick up her children from school and ended up showing her despair with tears in the middle of class. Embarrassed, Dr. Alice skipped the next class and did not have the courage to continue the course. However, Dr. Esther called her house to see if she was okay, and at the next time they met in person, Dr. Ester comforted Dr. Alice saying that there was nothing wrong with what happened and that she should prioritize her children. Dr. Esther shared her personal struggles with having a family and a career and told Dr. Alice how she had not been fully devoted to motherhood precisely because of her work. Dr. Alice has always seen herself more as a professional than a family person, but her children say otherwise and that she was always very present. This shows how well Dr. Alice not only achieved professional success and became a reference in forensics but was also successful in family life.

Before finishing her master’s degree in 1987, Dr. Alice was nominated by Dr. Pouchet-Campos to teach in the private school Oswaldo Cruz Faculties (FOC) in São Paulo. In 1997, Dr. Alice finished her doctorate at the USP with the thesis entitled *“*Cocaine and Cocaethylene: Influence of Ethanol in postmortem concentrations of Cocaine”. During her doctorate, she spent a year and a half at the Utah State University in the USA working under Dr. Roger Foltz, whom she describes as a “life mentor”. During her time in Utah, accompanied by her husband and children, she discovered her passion for nature and met many professionals who had an impact on her. Among these people, Dr. Vina Spiehler, who presented herself as a “tough woman”, and Dr. Alice says that she used to associate “tough” with professional. However, despite Dr. Spiehler being a serious professional with whom Dr. Alice identified deeply, she was extremely sensitive, an opera lover, and very respectful of the Latin culture. When this episode happened, Dr. Foltz expressed his opinion that Dr. Alice was the opposite of Dr. Spiehler, that is, docile and sweet, which Dr. Alice did not agree and found it funny.

Dr. Alice says that during her career she never felt left aside for being a woman. At home, she was not treated differently for being a woman and she believes that this made her “behave as a man” professionally always standing out for her intelligence. Being hired in a public job, as she did at the IML, depends only on one's exam grade and not on the profile, race, gender, or age. Thus, being a woman was never something she felt as a difficulty in the IML. As a matter of fact, in some public agencies there are more women and black people than men and white people. Also, when she later began working in the private sector at the FOC, Dr. Alice did not experienced sexism of any kind, especially since Dr. Pouchet-Campos was the director of the college and did not distinguish people by their profile and considered only the qualifications of the candidates.

Dr. Alice says she only realized lately that she was often the only woman in leadership positions. Even though toxicology in Brazil is an area of science with a predominance of women, Dr. Alice understands today that she suffered gender discrimination during her career, but she was not aware of it at the time.

Dr. Alice was Secretary (1998) and President (2004) of the BSTox and was always active sharing the society with others: “wherever I go, I take toxicology with me”, she says. She has always been concerned with the SBTox's lack of political strength and the fact that the society does not offer “an expert training and title”. Thus, during her presidency years in the SBTox she ensured that the society was granted political voice and position in various public agencies, one of them being the National Health Surveillance Agency (ANVISA). As a matter of fact, Dr. Silvia Barros describes Dr. Alice as a “toxicology warrior”.

She is currently a Full Professor of Toxicology and Coordinator of the FOC Graduate School, and was a Professor of Forensic Toxicology at the Civil Police Academy of São Paulo, where she worked intensely from 2005 to 2014. She was a Brazil representative in TIAFT from 1995 to 2018, a member of the BSTox and the American Academy of Forensic Sciences (AAFS), and was conferred a Drug Analysis specialist by the United Nations Narcotics Division in 1994. She won six awards, including the Esther de Camargo Fonseca Moraes Medal, published 54 scientific articles, 8 books, and 20 book chapters.

Dr. Alice is also known for her enthusiasm for scientific dissemination and teaching. She says that “the more you give, the more you get” and she gave a lot during her career. Her contagious generosity inspires new generations of toxicologists, for whom she regrets not having better opportunities in Brazil currently. She is an expert in drug abuse, one of the pillars in forensic toxicology, and an exemplary woman.

## Dr. Gisela de Aragão Umbuzeiro

**Figure 4:**
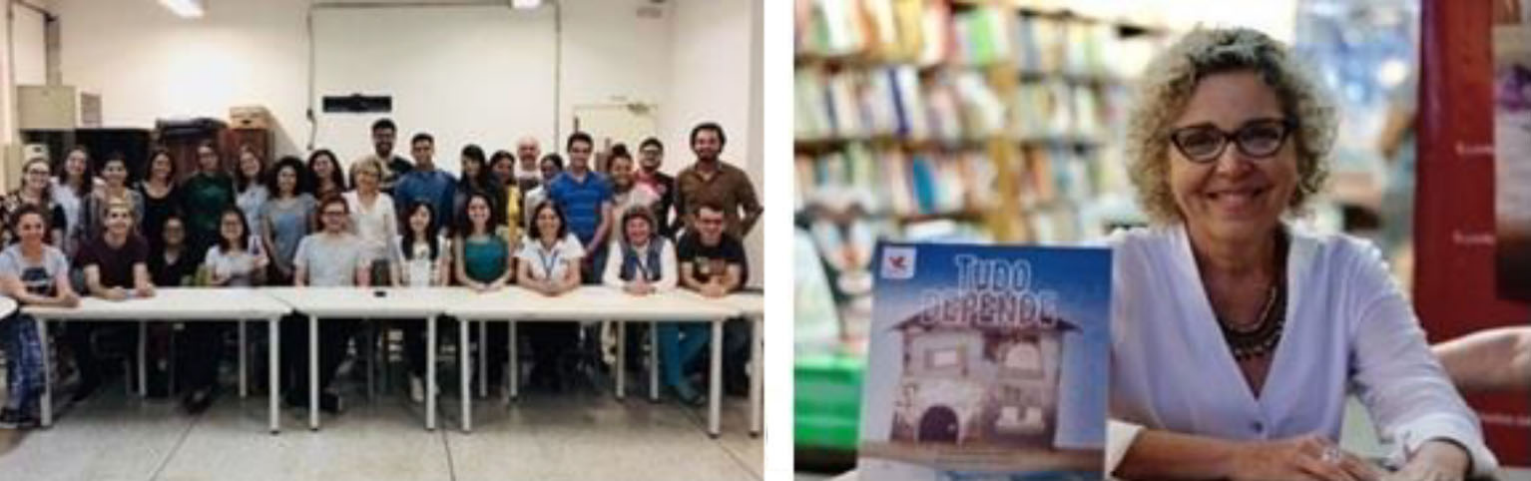
Dr. Gisela de Aragão Umbuzeiro with her students from University of Campinas (Unicamp) (left), and at the launch of her children's book “Tudo Depende” (right). Courtesy of Dr. Gisela de Aragão Umbuzeiro.

Born in Santos, a city on the coast of São Paulo State, Dr. Gisela (Figure 4) moved to Campinas at the age of 8 with her family because her father sought better work opportunities and better schools for his children.

In Campinas, Dr. Gisela studied at a technical school specializing in food technology, which made her fall in love with microbiology. Thus, she decided to apply for Chemistry and Agronomy major for college. However, because the technical school she studied put more emphasis on sciences than humanities, she did not score enough, and had to do a prep course, where she also worked, helping prepare classes.

In 1976, she started the Biology major in the University of Campinas (Unicamp). However, she was not satisfied with the major given that she was more interested in microbiology, and the program offered just one optional course in microbiology. Dr. Gisela was more interested in technology than in diseases or environment *per se*. She describes that her biggest dream at that time was to have a machine that she could use to see inside chromosomes. Therefore, she fell quickly in love with genetics after her first class of microorganism genetics with Dr. Renato Bonattelli Júnior, who at the time was doing his master's degree, and in the future become her master's and doctoral advisor.

At that time, she began working at the laboratory of filamentous fungi genetics, focusing on genetic improvement of specific strains for citric acid production and alcohol production from cassava. During her years as an undergraduate student, she dedicated herself to the laboratory and did not put much attention on the courses. Dr. Gisela tells that she used to go the morning classes normally but skipped afternoon classes so she could stay in the lab. Because of this, her grades were not exceptional, but high enough to get her into the next semester, and one day a professor told her that she was “great, but she was not born to be a biologist”.

She was a lab rat at that time giving such dedication to her experiments and was considered “overbearing” by some people given that she made a point of having her own projects inside the laboratory, and not just do what she was asked to in someone else's project. Without really realizing the importance of the work she carried out in the laboratory, her experiments were part of great discoveries, one of which was the report of a new reproductive process in filamentous fungi, called parasexuality.

In her senior year of college, for personal reasons, she finished her undergraduate course at the Federal University of Pernambuco. After that, in 1981 she returned to Campinas to do her master's degree in the same laboratory she previously worked in. It took longer than usual for her to get her master's degree, since during this period she had two daughters.

The master's years are described by her as years of “great determination and self-strength”. At that time, she lived in São Paulo city and commuted more than one hour to Campinas several times a week to take her master's classes and do her experiments. An interesting and sad fact is that, given that her husband had a well-paying position, she was denied a scholarship, making everything harder. Even with her mother's help, managing two young daughters while doing her master's was challenging. Dr. Gisela says it was common that she had to travel by bus to Campinas. To do so she had to take a bus to the Anhanguera Bus Terminal, then catch another one to Campinas, and then ride to the campus, all while she was seven months pregnant. When her first daughter was born and she had to stay home, she took home the materials she needed from the lab and did some of her experiments in one of the bathrooms of the house with a simple lamp to illuminate it. This scenario may seem of great struggle to many, but Dr. Gisela describes it with great joy and kindness. She used to repeat to herself: “I want to do it, so I will do it”.

After receiving her master's degree in 1985 with the thesis entitled “Isolation and Genetic Analysis of Mutants with Alterations in Amyloglycosidase Production in Aspergillus” under the supervision of Dr. Renato Bonattelli, she thought about continuing in graduate school and doing a doctorate, but preferred finding a job first.

At that time there were no job opportunities in universities, and she spent a lot of time looking for a job without success. After a while she was offered three positions to choose from: as a technician in a clinical laboratory, as a biologist in a pharmaceutical company that produced antibiotics, and in the Environmental Company of the State of São Paulo (CETESB) to work in the genetics area. She opted for the CETESB offer given the proximity to where she lived.

In 1986, she began her doctorate at Unicamp in the same laboratory she completed her masters while working at CETESB. Her schedule during those years was strict. She worked until 11 am, went to Campinas to take classes from noon to 1 pm and then went back to CETESB to work at 2 pm. All this while managing her daughters' school pick-up time and arriving on time for all appointments. As a matter of fact, to do so, she bought a moped, which surprised everybody.

In 1990 she finished her PhD at Unicamp with the dissertation entitled “Evaluation of the Mutagenic Activity of Organic Extracts from Water Bodies in the State of São Paulo Using the Ames test” that overlapped with her work at CETESB. Her work was pioneer in the area since the Ames test was not yet done in Brazil, especially in water samples.

Toxicology was never officially presented to Dr. Gisela, but she always understood the overlap with what she did and studied toxicology on her own. Over time, she became specialized and today she is considered a regulatory environmental toxicologist specialist.

From 2002 to 2005, she represented the CETESB in the CONAMA (National Environment Council, Brazil) 357 resolution, which regulates the use of water in Brazil. With the chance of participating in this resolution, she had to study and specialize herself in water quality for human and animal consumption and for aquatic life. Dr. Gisela has always been known for her reading abilities and it was common for her to read everything that was published and all reports from all environmental protection agencies in the world to understand a specific subject. She was also known for her firmness when debating and used to carry a green suitcase (before the advent of laptop computers) into meetings with all the articles, guidelines, reports, and regulations about the specific subject, and many envied her for having all the material available about the subject being discussed.

Dr. Gisela became a reference in her area. In one of her studies, she identified mutagenic dyes in the water that served the city of Cajamar, in São Paulo state, that could cause adverse effects in humans. Because of this, the river that supplied the city was diverted, preventing the exposure of more than 60,000 people to the mutagenic dyes.

Even with such influence and pioneering approach, Dr. Gisela did not stop. At the age of 52, she was hired at Unicamp as a professor. She believed that she had already fulfilled her mission at CETESB and resigned to take a chance in academia. As a professor she had to learn more about algae daphnia and trophic levels, subjects that she would later teach and use in her research. She is a pioneer in environmental toxicology at Unicamp and teaches regulatory toxicology, now mandatory for Sanitation and Environmental Engineering majors.

Currently, Dr. Gisela coordinates the Laboratory of Ecotoxicology and Genotoxicity (LAEG) at Unicamp, conducting research on evaluation of ecotoxicological risk of chemical substances including dyes, nanomaterials, and pesticides. She developed new acute and chronic toxicity testing protocols with aquatic and genotoxicity organisms, with specialized technicians and a high quality laboratory system in place. The LAEG performs toxicity tests with freshwater and saltwater organisms, in addition to genotoxicity assays *in vivo* and *in vitro* (11).

Dr. Gisela is part of several international projects such as: BioColour Project, a research project to develop new solutions for the production, characterization, and application of bio-dyes (dyes with biological basis), and the Global Panel on the Chemical Pollution of the Environment, an international organization to combat chemical pollution in the ecosystem (11).

She also participates in the newly created Brazilian Water Research Center (BWRC), a water research center based at Unicamp and a partnership with SANASA (Society for Water Supply and Sanitation), with FAPESP funding for 10 years. With this project she intends to accomplish her dream to take science into public policies contributing to the improvement of the quality of Brazil's waters.

In addition to her scientific and academic work, she also made important contributions in scientific dissemination and education. She is the author, together with her daughter Giovana, of a children's book called “It all depends” telling the story of Clarice, who observes and questions society's cultural habits. The story teaches children how to position themselves when facing contradictory issues. In addition to the book, she oversaw a dissertation entitled Development of an Educational Game on Pesticides in Food and Development of an Educational Kit for the Construction of Toxicology Concepts Using Experiment with Plants, showing Dr. Gisela's caring and compassionate side. As a matter of fact, her laboratory website is a great source of education materials for children (11).

Dr. Gisela had no perception that being a woman brought her any impediment or that she suffered any prejudice while studying, let alone in her family. Of her three siblings, she is the only a woman and was the best one in school. Thus, she was always seen and accepted as a person who liked to study, independently of her gender.

Dr. Gisela is a hurricane, always achieving what she wants considering what is best and right for the Brazilian community. More than a thousand students have passed through Dr. Gisela and all met her hard but loving personality. She stands out for her strength, and no pregnancy during graduate school, marriage, long and troubled commute between Campinas-São Paulo by bus or moped, or being denied a scholarship stopped her. Like she said herself, “I took a lemon and made orange juice”.

## Dr. Tania Marcourakis

**Figure 5:**
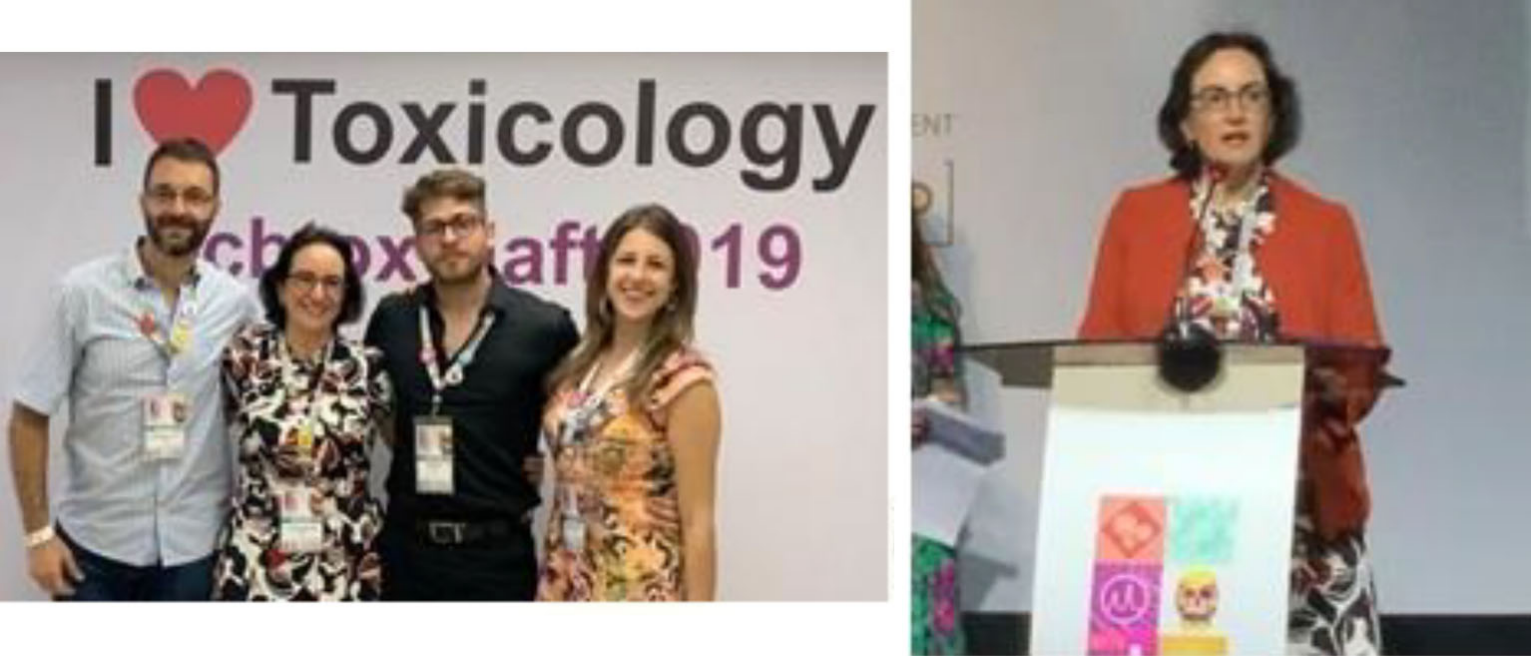
Dr. Tania Marcourakis with her students (left), and speaking at the XXI Brazilian Congress of Toxicology - XV TIAFT Latin-American Regional Meeting (right). Courtesy of Dr. Tania Marcourakis.

Dr. Tania (Figure 5) is the daughter of a Greek father and Jewish mother born in Egypt, who migrated to Brazil because she was expelled from Egypt in 1956 for being Jewish. Thus, both Dr. Tania and her sister were born in São Paulo as the first generation of Brazilians in the family. In Egypt, her father was a businessman from a family with resources, but when he moved to Brazil, he had to give up what he had and start over. In Brazil, Dr. Tania was raised in a Jewish community, where her uncles and aunts were the community people and not blood relatives.

Given the financial situation her family was in, she always attended public schools and knew that her only chance to get a college education was through a public university, more precisely the USP. Her parents always encouraged her to study and have good grades, as well as learning foreign languages. Also, her father always told her and her sister that they should be independent and never depend on husbands.

In 1980, she graduated from USP Pharmacy and Biochemistry School, specializing in clinical and toxicological analysis, but started working during her second semester at the age of 19 as a clinical analysis assistant at the Clinics Hospital of the Medical School of USP (HC-FMUSP), in the laboratory of the Neurology Investigation Center. She was later promoted to a biologist position and worked there for 24 years, being responsible for the therapeutic monitoring of anticonvulsants, developing methods, and transferring results to physicians. Her working hours were not rigid, which gave her plenty of freedom to raise her daughters and to be a ‘present’ mother. However, the position had no room for advancement and the salary was not satisfactory.

During her years at the Clinics Hospital, Dr. Tania pursued graduate studies. In 1986, she began her master's degree in pharmacology at the Institute of Biomedical Sciences (ICB) of USP graduating in 1990 defending her thesis entitled “Effects of the Chronic Administration of Clomipramine and Imipramine on Psychomotor Performance and Memory of Patients with Panic Syndrome”, under supervision by Dr. Clarice Gorenstein, who also mentored her during her doctorate. Despite having graduated from ICB, her research was developed at the Institute of Psychiatry of HC-FMUSP because of bureaucratic reasons. In the beginning of Dr. Tania's master, Dr. Clarice could not yet be her mentor and Dr. Valentim Gentil Filho, a psychiatrist, head of the Anxiety Clinic, took over.

The time she spent with Dr. Valentim's laboratory group was very productive to her from the scientific point of view given that the group was very open to communication among members. Dr. Tania defended her doctorate in 1995 with the thesis entitled “Relationship between the Serum Level and Clinical Effect of Clomipramine in Panic Disorders and Obsessive-Compulsive Disorder”.

In 2002, Dr. Tania spent 3 months at the Mayo Clinic in Rochester, Minnesota, funded by FAPESP. Dr. Tania describes this period as very satisfying personally but not professionally. The laboratories in the United States are known for high-quality technology and easy access to resources, but the laboratory P.I. in which she was working did not offer her much professionally during her months there.

In 2005, Dr. Clarice told her about a job opening at the USP as a professor in toxicology. Although Dr. Tania was tired of going through unsuccessful applications, she tried anyway and got the job. Having worked for so long in therapeutic supervising, she was considered both a pharmacologist and a toxicologist.

When she became a professor, Dr. Tania had to reinvent herself. From the beginning of her carrier, she had no interest in animal models, but now in academia she had to familiarize herself to it. Along with some of her students, especially Larissa Helena Lobo Torres and Raphael Caio Tamborelli Garcia, she learned about cell culture and animal methods.

Because of her experience in neurodegenerative diseases, such as Alzheimer's disease, and oxidative stress, she then began her research career emphasizing neurotoxicity of cigarette smoke, methylecgonidine (a product of cocaine pyrolysis), and bisphenol-A, making use of *in vivo* and *in vitro* models.

Smoking is one of the main causes of chronic illness and it is estimated that 1.6 billion people will be smokers by 2030. The research developed by Dr. Tania has already made great contributions to a better understanding of the inflammatory response of the central nervous system when exposed to cigarette smoke. In addition, her most current FAPESP grant is focused on the effects of ayahuasca on the neuroplasticity modulation produced by cocaine administration in mice. Preliminary results showed that ayahuasca is able to inhibit the expression of cocaine-induced behavioral sensitization (12).

Dr. Tania, since joining the Faculty of Pharmaceutical Sciences (FCF)-USP, has always been dedicated to undergraduate education and has completed six years as President of the Undergraduate Committee of FCF-USP. She is also very active in organizing and participating in conferences, such as the USP Undergraduate Conference, in which she was a member of the scientific committee several times, and the 9th Congress of Toxicology in the Developing Countries - CTDC9/XIX Brazilian Toxicology Congress.

Dr. Tania says she suffered some disguised misogyny at times in her career, but never felt diminished by being a woman. Although she graduated with emphasis on toxicology, she had a pharmaceutical career and only later returned to toxicology. In 2018, Dr. Tania became president of the SBTox and believes that today the greatest challenge of toxicology in Brazil is to unite the different societies into one and join forces for a common good.

## Final Comments

This article was based on interviews and shares a summary of five female scientists who have made Brazilian toxicology as we know it today, each in her specific subarea of toxicology. When asked, all of them said they had not experienced gender discrimination in their entire professional life, but all of them told stories of overcoming and struggling to raise their sons and daughters while having to study and develop a career. Clearly, they all carried an extra load when compared to their male colleagues, but they were so focused on doing their best that they did not see it. All of them were or still are great names in science as well as loving mothers.

Another common feature is that all were encouraged to study and taught to be independent from an early age. They were taught that they could achieve whatever they wanted, so they never doubted that science was their place. They are examples of admirable struggle and persistence, and we hope this article will inspire future generations with their stories.

Giving better conditions for women to work and have children, longer paternity leave, and taking into consideration when hiring that some women may have given up opportunities because of motherhood may be some suggestions to equate careers between genders.
